# Acute Pulmonary Embolism Associated with Low-Dose Olanzapine in a Patient without Risk Factors for Venous Thromboembolism

**DOI:** 10.1155/2021/5138509

**Published:** 2021-07-27

**Authors:** Vu Hoang Vu, Nguyen Duong Khang, Mai Thanh Thao, Le Minh Khoi

**Affiliations:** ^1^Cardiovascular Center, University Medical Center, University of Medicine and Pharmacy at Ho Chi Minh City, Vietnam; ^2^Department of Radiology, University Medical Center, Ho Chi Minh City, Vietnam

## Abstract

**Background:**

Olanzapine is a second-generation antipsychotic drug commonly prescribed for certain mental/mood conditions such as schizophrenia and bipolar disorders. This agent has been considered a precipitating factor for venous thromboembolism formation. Most of the cases previously reported were associated with high-dose olanzapine therapy or in patients with high-risk factors for the development of thromboembolism. *Case Presentation*. We report a patient who developed pulmonary embolism after a long course of low-dose olanzapine. A 66-year-old female patient suffering from insomnia had been prescribed olanzapine 2.5 mg and paroxetine 10 mg for two years. The patient suddenly developed a syncopal episode at home and was immediately brought to the hospital. The diagnosis of pulmonary embolism was made by chance during the computerized tomography of coronary arteries. The patient made a full recovery under conventional treatment and was discharged in stable condition. The thoracic computed tomography taken two months after discharge showed a completely normal pulmonary arterial tree.

**Conclusion:**

Olanzapine-associated pulmonary embolism is a rare entity and might be missed if the physician in charge is not vigilant and well informed. Even low-dose olanzapine can be associated with pulmonary embolism in patients with low classic risk factors if the treatment is prolonged. Pulmonary embolism should be sought in patients taking olanzapine even though the presenting manifestations are nonspecific.

## 1. Introduction

Venous thromboembolism is widely known as a common clinical entity with well-established classic risk factors, including but not limited to immobilization for more than three days, pregnancy, lower limb fracture, infection, cancer, and some medications. Olanzapine, a second-generation antipsychotic agent widely prescribed for patients with schizophrenia, has been recognized as a precipitating factor for venous thromboembolism [1, 2]. Most of the previous cases had been associated with high-dose olanzapine therapy or in patients with high-risk factors for the development of thromboembolism. We reported a patient without classic risk factors who developed symptomatic pulmonary embolism (PE) after being treated with low-dose olanzapine for insomnia.

## 2. Case Presentation

A 66-year-old female patient without a prior cardiovascular condition suddenly experienced a collapse for the first time and was reportedly unconscious for around 10 minutes. No abnormalities in facial expressions, limb weakness, drooling, or chest discomfort were noted during the crisis.

**Figure 1:**
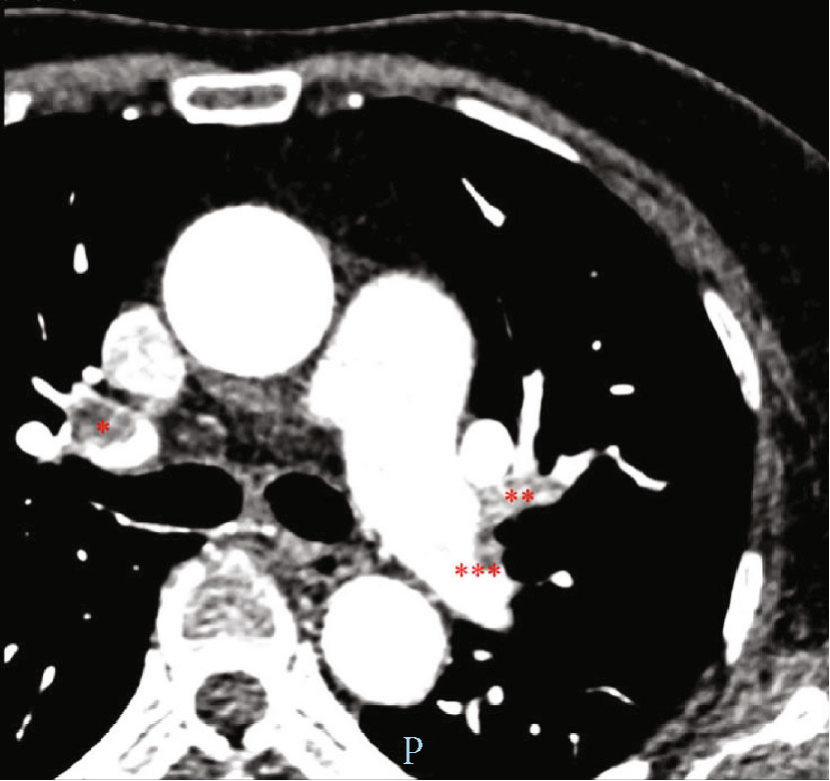
The spiral computed tomography angiogram obtained with highly concentrated contrast material and high flow technique showed multiple pulmonary emboli that subtotally occluded the right upper lobar pulmonary artery (^∗^) and the left lingular artery (^∗∗^). The left lower lobar pulmonary artery was also partially occluded (^∗∗∗^).

**Figure 2:**
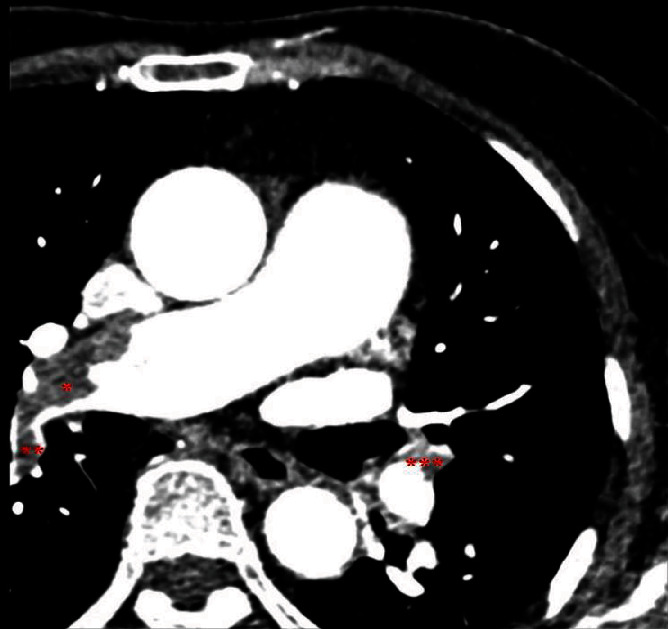
The spiral computed tomography angiogram obtained with highly concentrated contrast material and high flow technique showed multiple pulmonary emboli that partially occluded the right lower lobar pulmonary artery (^∗^), the right lower lobar superior/apical segment (S6), and the left lower lobar anteromedial segment (S7-8) (^∗∗∗^).

On admission to the emergency department, she was afebrile, alert, normotensive with a blood pressure of 135/82 mm Hg, a heart rate of 76 bpm, and nonobese with a BMI of 24.2 kg/m^2^. Her oxygen saturation was 87% on room air. Physical examination showed no noticeable abnormalities. Complete blood cell count was in the normal range. Blood biochemistry, including blood urea nitrogen, creatinine, high-sensitive troponin T, and N-terminal pro-BNP, was all in the normal range. The non-ST elevated myocardial infarction was excluded. Due to the syncopal episode at home, a head computed tomography was then performed, showing an extradural hematoma due to the collapse, but no other cerebral malformation was noted. We decided to have a coronary computed tomography angiography performed that ruled out coronary artery disease but revealed a pulmonary embolism. Multiple emboli were noted, causing a subtotal occlusion of the distal segment of the right pulmonary artery (Figure 1). The left pulmonary artery was also partially occluded (Figure 2). A venous duplex scanning for proximal deep vein thrombosis was performed that revealed no venous clot. Due to the presence of PE, we proceeded to screen malignant diseases and autoimmune diseases and test the autoimmune antibodies and possible causes of hypercoagulopathy, which showed no abnormality. Retrospectively, we found that the patient had been taking olanzapine 2.5 mg daily for the previous two years for her insomnia. Olanzapine was then discontinued on the first day of hospitalization.

**Figure 3:**
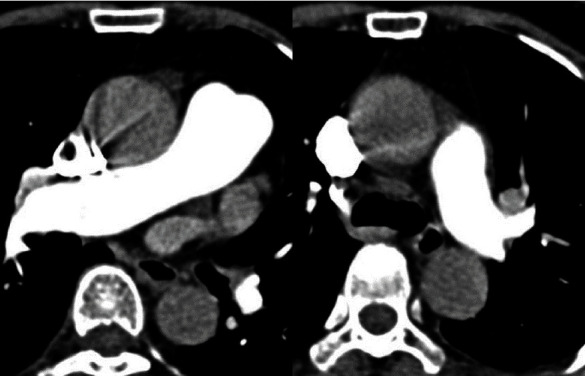
The spiral computed tomography angiogram obtained with highly concentrated contrast material and high flow technique showed that the contrast material filled the right pulmonary arterial system, and there was no embolus as previously documented in Figure 2.

With the pulmonary embolism severity index (PESI) score of 66 points and the presence of an epidural hematoma, we decided to start continuous intravenous heparin for the first two days, followed by low molecular weight heparin for the next three days, and finally oral dabigatran 150 mg twice a day. The patient was discharged three weeks after the onset and has been regularly followed up at the outpatient clinic. During the follow-up period, she experienced no remarkable events. A computed tomographic pulmonary angiography ordered two months after her discharge showed complete resolution of the previously documented PE in the pulmonary arterial system (Figure 3). At the time of preparing this manuscript, the patient is still on oral Dabigatran.

## 3. Discussion

Olanzapine is a second-generation antipsychotic agent commonly prescribed for schizophrenia. Its typical side effects include weight gain, drowsiness, and other antimuscarinic effects. The association between antipsychotic agents and venous thromboembolism was first described in the 1950s [3]. Wagge and Gedde-Dahl [4] reported a clinical case of a 28-year-old male patient who developed after starting treatment with high-dose olanzapine. A case series also described the possible association between olanzapine and venous thromboembolism after a 2-week course of treatment in four patients. The doses of olanzapine in these patients ranged between 5 and 20 mg per day and lasted from seven weeks to 17 months [5, 6]. In clinical practice, if the physicians at the emergency department are not well informed, they may miss the early diagnosis of PE in patients taking olanzapine. Our emergency physician could not suspect the diagnosis of PE and did not proceed with the PE protocol by our institutional PE response team (PERT). The PE was incidentally established upon the thoracic CT to investigate the coronary artery system.

The mechanism by which olanzapine triggers or precipitates the thromboembolic conditions remains unknown. The olanzapine-induced metabolic changes such as dyslipidaemias, hyperleptinemia, hyperglycaemia, and hyperhomocysteinemia might, in part, account for a decreased thrombolysis but hardly responsible for the venous thromboembolism events [3]. Olanzapine is a 5-hydroxytryptamine receptor antagonist. Therefore, it may raise the blood serotonin leading to accelerated platelet aggregation and increased thromboembolic risk.

Our patient did not present any classic thrombotic risk factors (e.g., immobility, fracture, myocardial infarction, history of previous thromboembolism, malignant diseases, and hypercoagulable conditions). Thereby, we strongly suspected that it was associated with olanzapine. Of importance, the olanzapine dose in this patient was considerably low, only at 2.5 mg daily. Some case reports did recognize the thromboembolic events in patients taking olanzapine. However, the lowest dose reported in these observations was 5 mg per day. Like our case, these reports did not document any other thrombotic risk factors, except obesity in two patients. Our observation would emphasize the association between the oral olanzapine, even at a low dose, and thromboembolic events.

The management of olanzapine-associated venous thromboembolism consists of olanzapine discontinuation and guideline-based anticoagulation [7]. Our patient had a PESI score of 66 and an epidural hematoma secondary to falling at home; therefore, we decided to start continuous intravenous heparin but not thrombolytic therapy. This therapeutic approach proved effective: the patient became asymptomatic, and the pulmonary arterial tree was cleared of thrombus.

## 4. Conclusion

Olanzapine-associated is a rare entity and might be missed if the physician in charge is not vigilant and well informed. Our report emphasized that even low-dose olanzapine can be associated with if the treatment is prolonged. Deep vein thrombosis and pulmonary embolism should be sought in patients taking olanzapine even though the presenting manifestations are atypical.

## Data Availability

The datasets used and/or analysed during the current study are available from the corresponding author on reasonable request.

## References

[1] Barbui C., Conti V., Cipriani A. (2014). Antipsychotic drug exposure and risk of venous thromboembolism: a systematic review and meta-analysis of observational studies. *Drug Safety*.

[2] Hägg S., Bate A., Stahl M., Spigset O. (2008). Associations between venous thromboembolism and antipsychotics. A study of the WHO database of adverse drug reactions. *Drug Safety*.

[3] Liperoti R., Pedone C., Lapane K. L., Mor V., Bernabei R., Gambassi G. (2005). Venous thromboembolism among elderly patients treated with atypical and conventional antipsychotic agents. *Archives of Internal Medicine*.

[4] Waage I. M., Gedde-Dahl A. (2003). Pulmonary embolism possibly associated with olanzapine treatment. *BMJ*.

[5] Maly R., Masopust J., Hosak L., Urban A. (2009). Four cases of venous thromboembolism associated with olanzapine. *Psychiatry and Clinical Neurosciences*.

[6] Hägg T., Tätting P., Spigset O. (2003). Olanzapine and venous thromboembolism. *International Clinical Psychopharmacology*.

[7] Konstantinides S. V., Meyer G., Becattini C. (2020). 2019 ESC Guidelines for the diagnosis and management of acute pulmonary embolism developed in collaboration with the European Respiratory Society (ERS). *European Heart Journal*.

